# Evolution de la résistance aux antibiotiques des entérobactéries isolées à l'Hôpital Général de Douala de 2005 à 2012

**DOI:** 10.11604/pamj.2015.20.227.4770

**Published:** 2015-03-12

**Authors:** Cécile Okalla Ebongue, Martial Dongmo Tsiazok, Jean Pierre Nda Mefo'o, Guy Pascal Ngaba, Gérard Beyiha, Dieudonné Adiogo

**Affiliations:** 1 Laboratoire de Biologie Clinique de l'Hôpital Général de Douala, Douala, Cameroun; 2 Département des Sciences Biologiques, Faculté de Médecine et des Sciences Pharmaceutiques, Université de Douala, Douala, Cameroun; 3 Service de Réanimation de l'Hôpital Général de Douala, Douala, Cameroun

**Keywords:** Cameroun, entérobactéries, résistance aux antibiotiques, Cameroon, Enterobacteriaceae, antibiotic resistance

## Abstract

**Introduction:**

Cette étude vise à déterminer le profil de résistance aux antibiotiques des entérobactéries isolées à l'Hôpital Général de Douala (Cameroun) et analyser leur évolution dans le temps.

**Méthodes:**

Etude rétrospective, sur une période de huit ans (2005 - 2012), portant sur l'ensemble des souches d'entérobactéries isolées chez les malades ambulatoires et hospitalisés. Les prélèvements ont été analysés au laboratoire de bactériologie de l'Hôpital Général de Douala.

**Résultats:**

Les entérobactéries étaient les germes les plus fréquents sur l'ensemble des souches isolées. Nous avons noté une prédominance d’*Escherichia coli* (48,5%) et de *Klebsiella pneumoniae* (32,8%). Pendant la période d’étude, nous avons observé des taux de résistance élevés aux principales classes d'antibiotiques, et une augmentation entre 2005 et 2012 de 29,1% à 51,6% pour les céphalosporines de troisième génération, de 29,2% à 44% pour la ciprofloxacine. L'imipénème, l'amikacine et la fosfomycine étaient les molécules les plus actives avec respectivement 1,3%, 12,9% et 13,4% des souches d'entérobactéries résistantes.

**Conclusion:**

L’évolution des résistances des entérobactéries aux antibiotiques est un phénomène réel dans la ville de Douala. Il expose à des difficultés de prise en charge thérapeutique des infections. Lamaitrise actuelle de ce phénomène est une véritable urgence et nécessite une implication des pouvoirs publics. Des tests spécifiques de recherche des bétalactamases à spectre élargi (BLSE) et AmpC doivent être mis en place dans nos laboratoires afin de mettre en évidence les différents phénotypes de résistances.

## Introduction

La résistance bactérienne aux agents antimicrobiens est un problème d'importance croissante en pratique médicale [1, 2]. La dissémination des bactéries résistantes est à l'origine d'une augmentation considérable de la mortalité, de la morbidité ainsi que du coût des traitements [3, 4]. Parmi les germes responsables d'infections bactériennes, les entérobactéries sont les plus redoutables car elles sont productrices de bétalactamases et possèdent d'autres mécanismes de résistanceà de nombreux antibiotiques [5–7]. La concentration importante de ces germes dans le tube digestif, favorise l’échange et la dissémination des gènes de résistance [8, 9].

Cette résistance bactérienne aux antibiotiques pose le problème de choix de l'antibiothérapie. En Afrique, et en particulier au Cameroun, la majorité des patients n'ont pas accès au laboratoire et la prise en charge des syndromes infectieux est probabiliste. L'efficacité de cette approche réside dans la connaissance de l’écologie microbienne locale, et de l’évolution des résistances des bactéries aux agents anti microbiens. Des études faites au Cameroun ont montré la présence d'entérobactéries productrices de bétalactamases à spectre élargi dans les villes de Yaoundé et NGaoundéré [10, 11]. L'objectif de ce travailest d'identifier les souches d'entérobactéries isolées au laboratoire de l'Hôpital Général de Douala (HGD) au Cameroun de 2005 à 2012, et d’étudier l’évolution de leurs profils de résistance aux antibiotiques.

## Méthodes

### Lieu et type d’étude

Il s'agit d'une étude rétrospective, descriptive menée au laboratoire de biologie clinique de l'HGD sur une période de huit ans, du 1er janvier 2005 au 31 décembre 2012. L'HGD est une structure sanitaire publique de référencesituée dans la région du Littoral, et comptant 210 lits. Cet hôpital regroupe les services de médecine générale, gynécologie-obstétrique, pédiatrie et néonatalogie, chirurgie générale et orthopédie, réanimation, ainsi qu'une unité de prise en charge des grands brûlés et un service d'hémodialyse.

### Souches étudiées

L'ensemble des isolats d'entérobactéries provenant des prélèvements à visée diagnostique, des malades hospitalisés dans les différents services et des malades ambulatoires reçus au cours de la période d’étude ont été inclus.

### Identificationet étude de la sensibilité aux antibiotiques

Les prélèvements ont été mis en culture et incubés pendant 24 heures à 37°C, sur gélose Cystine Lactose Electrolyte Déficient (CLED) pour les urines, Hektoen pour les selles, et Eosine Bleu de méthylène (EMB) pour les autres (pus, liquides de ponctions, prélèvements génitaux, cathéters, sondes urinaires et hémocultures). Après observation macroscopique des colonies, l'identification des bactéries, fondée sur l’étude des caractères biochimiques et enzymatiques, a été faite par ensemencement des galeries ID32E^®^, et lecture colorimétrique automatique sur mini API^™^ (bio Mérieux SA, Marcy l’étoile, France) après 24 heures d'incubation.

L’étude de la sensibilité aux antibiotiques a été réalisée par la technique de dilution sur des galeries ATBGN^®^ et ATBUR^®^, suivie d'une incubation pendant 24 heures, puis lecture turbidi-néphélométrique automatiqueet expertise des résultats sur mini API^™^, selon les normes du comité de l'antibiogramme de la Société Française de Microbiologie [12]. Au total, 19 molécules d'antibiotiques ont été étudiées dans les galeries d'antibiogramme (amoxicilline; Amoxicilline + acide clavulanique;ticarcilline; pipéracilline; pipéracilline + tazobactam; céfalotine; céfoxitine; céfotaxime; ceftazidime; gentamicine; tobramycine; neltimicine; amikacine; norfloxacine; ofloxacine; ciprofloxacine; sulfaméthoxazole + Triméthoprime; fosfomycine; imipénème).

### Collecte des données

Elle a consisté en l'extraction des résultats de tous les antibiogrammes réalisés de 2005 à 2012 enregistrés dans la mémoire de l'automate mini API^™^, grâce à une disquette 3 ½ (sur format Excel). Ces résultats comportaient, le numéro d'identification, le nom du patient, la date d'isolement, le germe identifié, la nature du prélèvement, ainsi que les antibiotiques testés avec leur profil de sensibilité (S, I, R). Ces données ont été complétées avec la provenance du prélèvement grâce aux registres du laboratoire.

### Traitement des données et analyse statistique

Dans l’établissement des pourcentages de résistance desdifférentes espèces bactériennes, les résultats « intermédiaire » ont été inclus dans la catégorie « résistant ». L'analyse descriptive des données a été faite à l'aide des logiciels EPI Info version 3.5 et Microsoft Excel 2007. Les proportions ont été comparées par le test de chi carré. Une valeur de p < 0,05 a été considérée comme statistiquement significative. Limites: dans cette étude, l'interprétation des profils de résistance a été faite par le système expert de l'automate mini Api^™^, mais n'a pas été systématiquement confirmée par des tests spécifiques. Par ailleurs, la notion de prise préalable d'antibiotiques par les malades avant la réalisation des examens bactériologiques n’‘était pas connue.

## Résultats

### Etude des souches isolées

Au cours de la période d’étude, un total de 4497 souches ont été isolées de tous les prélèvements reçus au laboratoire de bactériologie de l'Hôpital Général de Douala. Les entérobactéries représentaient 71% de l'ensemble des germes isolés, soit 3195 souches. Parmi ces souches, 656 provenaient du service de médecine, 535 de pédiatrie, 315 de chirurgie, 209 de réanimation, 132 de gynécologie et 35de néonatologie. Les souches provenant des prélèvements des malades externes représentaient41,1% de l'ensemble des isolats d'entérobactéries (1313 souches).

*Escherichia coli* était l'espèce la plus fréquemment isolée (48,5%), suivie de *Klebsiella pneumoniae* (32,8%) et Enterobacter cloacae (4,2%). En plus des espèces couramment rencontrées dans les infections (*Proteus, Citrobacter, Enterobacter, Morganella, Providencia, Salmonella, Serratia et Shigella*), ont également été isolées en très faible proportion des espèces plus rares comme *Raoultella spp* (5 souches des suppurations et des urines), *Edwarsiella tarda* (1 souche des urines), *Hafniaalvei* (2 souches des urines), *Pantoea spp* (7 souches des urines, des suppurations, et hémocultures).

La majorité des souches provenait des urines(2196 soit 68,7%), des suppurations(433 soit 13,5%) et des hémocultures (220 soit 6,9%). Dans les selles seules *Salmonella spp*et *Shigella spp* ont été isolées comme bactéries entéropathogènes. Suivant les années, 101 souches ont été isolées en 2005, 224 en 2006, 186 en 2007, 370 en 2008, 458 en 2009, 526 en 2010, 778 en 2011, et 553 en 2012.

### Etude de la sensibilité aux antibiotiques

#### Bétalactamines

La résistance des souches aux aminopénicillines, qu'elle soit naturelle ou acquise était élevée (93,1%). En présence d'acide clavulanique, cette résistance baissait pour atteindre le taux de 75,2%. L'association pipéracilline + tazobactam était un peu plus active avec 39,1% des souches résistantes (Tableau 1).

**Table 1:** Profil de résistance des entérobactéries dans les différents prélèvements

	Cathéter n / N (%)	Liquide de ponction n / N (%)	Prélèvement génital n / N (%)	Pus n / N (%)	Sang n / N (%)	Selles n / N (%)	Sonde urinaire n / N (%)	Urine n / N (%)	Total n / N (%)
AMO	51 / 53 (96,2)	15 / 16 (93,8)	10 /12 (83,3)	419 / 428 (97,9)	199 / 218 (91,3)	46 / 86 (53,5)	168 / 173 (97,1)	2022 / 2162 (93,5)	2930 / 3148 (93,1)
AMC	44 / 52 (84,6)	15 / 16 (93,8)	8 / 12 (66,7)	371 / 428 (86,7)	173 / 217 (79,7)	29 / 86 (33,7)	156 / 175 (89,1)	1575 / 2167 (72,7)	2371 / 3153 (75,2)
TIC	50 / 54 (92,6)	14 / 16 (87,5)	9 / 11 (81,8)	387 / 427 (90,6)	185 / 218 (84,9)	42 / 85 (49,4)	160 / 172 (93)	1890 / 2118 (89,2)	2737 / 3101 (88,3)
PIC	50 / 54 (92,6)	13 / 15 (86,7)	8 / 10 (80)	363 / 397 (91,4)	175 / 203 (86,2)	40 / 83 (48,2)	128 / 135 (94,8)	928 / 1006 (92,2)	1705 / 1903 (89,6)
TZP	26 / 51(51)	7 / 15 (46,7)	1 / 10 (10)	156 / 391 (39,9)	78 / 200 (39)	3 / 80 (3,8)	66 / 135 (48,9)	351 / 878 (40)	688 / 1760 (39,1)
CFT	48 / 52 (92,3)	16 / 16 (100)	7 /10 (70)	376 / 420 (89,5)	178 / 211 (84,4)	24 / 83 (28,9)	157 / 172 (91,3)	1687 / 2135 (79)	2493 / 3099 (80,4)
CXT	24 / 52(46,2)	6 / 16 (37,5)	1 / 7 (14,3)	174 / 400 (43,5)	96 / 215 (44,7)	4 / 83 (4,8)	72 / 167 (43,1)	507 / 1952 (26)	884 / 2892 (30,6)
CTX	43 / 54 (79,6)	11 / 16 (68,8)	3 / 10 (30)	282 / 428 (65,9)	142 / 217 (65,4)	4 / 86 (4,7)	133 / 173 (76,9)	813 / 2165 (37,6)	1431 / 3149 (45,4)
CAZ	43 / 54 (79,6)	10 / 16 (62,5)	4 / 11 (36,4)	273 / 427 (63,9)	140 / 217 (64,5)	4 / 86 (4,7)	130 / 173 (75,1)	800 / 2163 (37)	1404 / 3147 (44,6)
GEN	38 / 54 (70,4)	10 / 16 (62,5)	7 / 12 (58,3)	272 / 429 (63,4)	126 / 218 (57,8)	4 / 86 (4,7)	121 / 175 (69,1)	842 / 2169 (38,8)	1420 / 3159 (45)
TOB	39 / 54 (72,2)	12 / 16 (75)	6 / 12 (50)	250 / 429 (58,3)	119 / 218 (54,6)	3 / 86 (3,5)	120 / 175 (68,6)	830 / 2165 (38,3)	1379 / 3155 (43,7)
NET	14 / 21 (66,7)	8 / 12 (66,7)	5 / 12 (41,7)	135 / 265 (50,9)	65 / 138 (47,1)	1 / 55 (1,8)	69 / 108 (63,9)	460 / 1463 (31,4)	757 / 2074 (36,5)
AKN	12 / 54 (22,2)	6 / 16 (37,5)	3 / 12 (25)	61 / 427 (14,3)	29 / 218 (13,3)	1 / 86 (1,2)	34 / 176 (19,3)	262 / 2169 (12,1)	408 / 3158 (12,9)
NOR	5 / 7 (71,4)	3 / 4 (75)	2 / 5 (40)	42 / 73 (57,5)	14 / 29 (48,3)	1 / 14 (7,1)	42 / 62 (67,7	755 / 1748 (43,2)	864 / 1942 (44,5)
OFL	36 / 52 (69,2)	10 / 15 (66,7)	3 / 6 (50)	238 / 393 (60,6)	99 / 210 (47,1)	3 / 82 (3,7)	104 / 166 (62,7)	839 / 1899 (44,2)	1332 / 2823 (47,2)
CIP	33 / 54 (61,1)	11 / 16 (68,8)	5 / 11 (45,5)	225 / 423 (53,2)	95 / 218 (43,6)	2 / 84 (2,4)	104 / 175 (59,4)	804 / 2149 (37,4)	1279 / 3130 (40,9)
TSU	46 / 54 (85,2)	13 / 16 (81,3)	11 / 11 (100)	346 / 428 (80,8)	166 / 218 (76,1)	49 / 85 (57,6)	145 / 175 (82,9)	1768 / 2167 (81,6)	2544 / 3154 (80,7)
FOS	12 / 49 (24,5)	2 / 15 (13,3)	1 / 5 (20)	87 / 383 (22,7)	39 / 208 (18,8)	1 / 82 (1,2)	29 / 163 (17,8)	219 / 1997 (11)	390 / 2902 (13,4)
IMI	0 / 54 (0,0)	0 / 15 (0,0)	2 / 12 (16,7)	6 / 416 (1,4)	3 / 209 (1,4)	0 / 85 (0,0)	3 / 143 (2,1)	11 / 937 (1,2)	25 / 1871 (1,3)

n = nombre de souches résistantes ; N = nombre de souches testées à l'antibiotique. AMO: amoxicilline; AMC : Amoxicilline + acide clavulanique ; TIC: ticarcilline; PIC: pipéracilline; TZP : pipéracilline + tazobactam ; CFT: céfalotine; CXT : céfoxitine ; CTX : céfotaxime ; CAZ : ceftazidime ; GEN : gentamicine ; TOB : tobramycine ; NET : neltimicine ; AKN : amikacine ; NOR : norfloxacine ; OFL : ofloxacine ; CIP:ciprofloxacine ; TSU: sulfaméthoxazole + Triméthoprime ; FOS : fosfomycine ; IMI : imipénème

Au total 1394 (44%) souches ont montré un phénotype de résistance aux céphalosporines de troisième génération (C3GR), avec comme principales espèces *Klebsiella pneumoniae* (680 souches), *Escherichia coli* (502 souches), *Enterobacter cloacae* (83 souches), *Enterobacter* autre que *E. cloacae* (45 souches), *Morganellamorganni* (17 souches), *Proteus mirabilis* et *Serratia spp* (15 souches chacune), *Citrobacter spp* (14 souches), *Providencia spp* (10 souches).

La majorité des souches C3GR ont été isolées des urines (795), suivies des suppurations (271), des hémocultures (138), des sondes urinaire (130), des cathéters (43) et des liquides de ponction (10). Parmi ces souches 395 provenaient des prélèvements des patientsambulatoires (28,3%). Selon la provenance des prélèvements au sein de l'hôpital, la résistance aux C3G (céfotaxime et ceftazidime) était plus élevée dans les services de néonatologie (85,7% pour les 2 molécules), de réanimation (79,4% pour le céfotaxime et 77,9%pour la ceftazidime) et de chirurgie (74,5% et 72,2% respectivement pour le céfotaxime et la ceftazidime) que dans les autres services.

**Figure 1:**
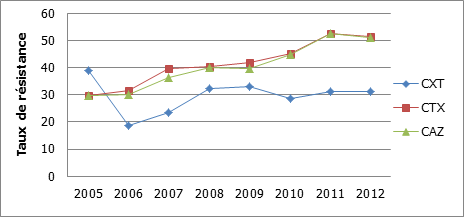
Evolution des résistances des entérobactéries aux céphalosporines CXT: céfoxitine; CTX: céfotaxime; CAZ: ceftazidime

Par rapport à 2005, cette résistance au céfotaxime et à la ceftazidime avait progressé les années suivantes de manière continue et identique pour les deux molécules (Figure 1). Par contre, la résistance à la céfoxitine avait baissé après 2005 et était devenue stable à partir de 2008 (Figure 1). Globalement, la céfoxitine était plus active que les C3G (Tableau 1).

#### Aminosides

**Figure 2:**
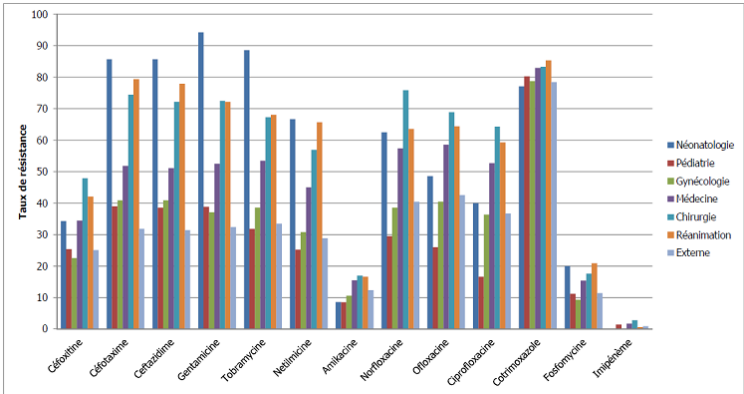
Profil de résistance des entérobactéries dans les services

**Figure 3:**
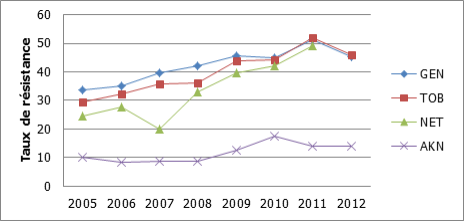
Evolution des résistances des entérobactéries aux aminosides GEN: gentamicine; TOB: tobramycine; NET: neltimicine; AKN: amikacine

Les aminosides présentaient une faible activité sur les souches isolées: gentamicine (94,3% / 72,1% / 72,5% de souches résistantes), tobramycine (88,6% / 68,1% / 67,3% de souches résistantes), néltimicine (66,7% / 65,7% / 56,9% de souches résistantes) respectivement dans les services de néonatologie, réanimation, et chirurgie (Figure 2). La résistance à ces trois molécules a augmenté au fil des années avec un pic en 2011 (Figure 3). La résistance à l'amikacine, a évolué de 10,2% à 14,1% entre 2005 et 2012.

**Figure 4:**
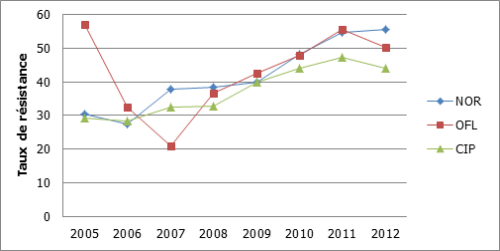
Evolution des résistances des entérobactéries aux quinolones NOR: norfloxacine; OFL: ofloxacine; CIP: ciprofloxacine

Quinolones: la ciprofloxacine était la quinolone la plus active pour l'ensemble des souches (Tableau 1). Dans les urines, on constatait que la résistance des entérobactéries aux fluoroquinolones était plus élevée que la résistance aux C3G (Tableau 1). La résistance aux quinolones a progressé de manière identique pour les 3 molécules (norfloxacine, ofloxacine et ciprofloxacine)testées (Figure 4).

**Figure 5:**
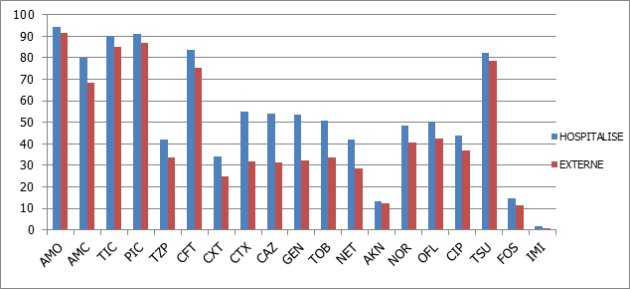
Comparaison des taux de résistance des souches d'entérobactéries chez les patients hospitalisés et non hospitalisés

D'un point de vue général, les souches isolées des prélèvements des patients hospitalisés étaient plus résistantes aux antibiotiques que celles des malades non hospitalisé. Ceci s'observe en particulier pour les C3G, les aminosides et les quinolones (Figure 5). Les molécules les plus actives sur les entérobactéries étaient l'imipenème avec un taux de résistance de 1,3%, suivie de l'amikacine (12,9%) et la fosfomycine (13,4%) (Tableau 1).

## Discussion

Les entérobactéries sont un groupe de bactéries fréquemment isolées dans les laboratoires de bactériologie, *E. coli* et *Klebsiella spp* étant les espèces revenant le plus souvent [13–16]. Dans la plupart des études, les souches isolées proviennent majoritairement des urines. Dans notre cas, ces isolats représentaient 68,7% de l'ensemble, comparable aux taux de 69,3% et 64,7% rapportés respectivement par Hashemi et al. en Iran [17] et Raji et al. au Nigéria [18].

Ces entérobactéries étaient hautement résistantes aux pénicillines et au cotrimoxazole. Au Cameroun Gangoué-Piéboji retrouvait 87% dessouches d'entérobactéries résistantes à l'amoxicilline et 73% de souches résistantes au cotrimoxazole [15]. Nous avons cependant noté l'activité supérieure de l'association pipéracilline + tazobactam qui restaurait plus l'activité des pénicillines que l'association amoxicilline + acide clavulanique. Ceci pouvant s'expliquer par le fait que l'association pipéracilline + tazobactam est peu utilisée à l'Hôpital Général de Doualaet qu'elle a été testée moins fréquemment que l'association amoxicilline + acide clavulanique. Des résultats similaires ont été retrouvés au Nigeria et en Espagne [18, 19]. Plus de 44% de résistance aux céphalosporines de troisième génération(céfotaxime et ceftazidime) a été observée, contrairement aux résultats del’étude deHashemiqui montraient une sensibilité de près de 75% [17]. En effet ces céphalosporines de troisième générationsont les antibiotiques les plus utilisés à l'HGD, notamment dans les services de néonatologie et de réanimation. D'autres auteurs ont fait un constat identique dans ces servicespar rapport à la moyenne de l'hôpital [20]. L'HGD étant une structure de référence, les patients viennent souvent d'autres formations sanitaires où des traitements probabilistes basés sur l'utilisation de ces molécules ont parfois déjà été initiés. L'automédication et l'absence de guideline de prise en charge des infections contribuent également à l'augmentation des niveaux de résistance à ces antibiotiques dans notre contexte.

La céfoxitine présentait une bonne activité sur les souches d'entérobactéries. Nous constatons une tendance régulière à l'augmentation des taux de résistance à la céfoxitine et aux C3G au fil des années. En Espagne et en Turquie, aucune évolution des résistances aux C3G n'a été retrouvée [19, 20]. Cette augmentation pourrait être due à la pression de sélection.

L'imipenème et l'amikacine avaient une bonne activité sur les souches d'entérobactéries. Cette tendance a également été retrouvée en Espagne [19]. Par contre Hashemi et al. retrouvaient des taux plus élevés de résistance de l'ordre de 19% pour l'imipénème et 30% pour l'amikacine [17]. L'amikacine qui était l'aminoside le plus actif sur les entérobactéries a présenté une baisse de son activité au fil des années. Cette molécule est de plus en plus utilisée à cause de l'inefficacité des autres aminosides (gentamicine, tobramycine et netilmicine), notamment dans les infections sévères en néonatalogie. Les souches d'entérobactéries avaient des taux élevés de résistance aux fluoroquinolones contrairement à l’étude de Hashemi en Iran [17]. Une progression du taux de résistance des entérobactéries à la ciprofloxacine a également été rapportée par Guembe en Espagne [19]. Cette résistance élevée dans notre étude trouve sa justification dans le fait que ces molécules sont les plus prescrites dans le traitement des infections urinaires. Ceci se confirme par des niveaux de résistance aux quinolones plus élevés dans les urines que les autres antibiotiques majeurs (céphalosporines et aminosides).

Bien qu'ayant noté des taux importants de résistance aux C3G pour les souches isolées des prélèvements des malades ambulatoires, l'ensemble des souches isolées chez les sujets hospitalisés était plus résistantes aux antibiotiques que celles des patients externes, constat identique rapporté par Hashemiet Piéboji [17, 21].

## Conclusion

Cette étude montre que le niveau de résistance des germes vis-à-vis des antibiotiques varie avec l'espace et le temps. Nous avons constaté entre 2005 et 2012, une augmentation dutauxde résistance aux antibiotiques des principaux germes isolés. Notre étude montre un niveau de résistance très élevé de nos souches vis-à-vis d'antibiotiques majeurs si on compare nos données à celles d'autres pays. Des souches présentant de haut niveau de résistance aux bétalactamines et au cotrimoxazole sont majoritaires. Seuls les carbapénèmes gardent une excellente activité. Des tests spécifiques de recherche des bétalactamases à spectre élargi (BLSE) et Amp C doivent être mis en place au sein du laboratoire, afin de mettre en évidence les différents phénotypes de résistance.
